# Behavioral Abnormalities Observed in *Zfhx2*-Deficient Mice

**DOI:** 10.1371/journal.pone.0053114

**Published:** 2012-12-31

**Authors:** Yuriko Komine, Keizo Takao, Tsuyoshi Miyakawa, Tetsuo Yamamori

**Affiliations:** 1 Division of Brain Biology, National Institute for Basic Biology, Okazaki, Japan; 2 Section of Behavior Patterns, National Institute for Physiological Sciences, Okazaki, Japan; 3 Genetic Engineering and Functional Genomics Group, Frontier Technology Center, Kyoto University Graduate School of Medicine, Kyoto, Japan; 4 Division of Systems Medical Science, Institute for Comprehensive Medical Science, Fujita Health University, Toyoake, Japan; 5 Core Research for Evolutional Science and Technology (CREST), Japan Science and Technology Agency (JST), Kawaguchi, Japan; University of Queensland, Australia

## Abstract

*Zfhx2* (also known as *zfh-5*) encodes a transcription factor containing three homeobox domains and 18 Zn-finger motifs. We have reported that *Zfhx2* mRNA is expressed mainly in differentiating neurons in the mouse brain and its expression level is negatively regulated by the antisense transcripts of *Zfhx2*. Although the expression profile of *Zfhx2* suggests that ZFHX2 might have a role in a particular step of neuronal differentiation, the specific function of the gene has not been determined. We generated a *Zfhx2*-deficient mouse line and performed a comprehensive battery of behavioral tests to elucidate the function of ZFHX2. Homozygous *Zfhx2*-deficient mice showed several behavioral abnormalities, namely, hyperactivity, enhanced depression-like behaviors, and an aberrantly altered anxiety-like phenotype. These behavioral phenotypes suggest that ZFHX2 might play roles in controlling emotional aspects through the function of monoaminergic neurons where ZFHX2 is expressed. Moreover, considering their phenotypes, the *Zfhx2*-deficient mice may provide a novel model of human psychiatric disorders.

## Introduction

**Figure 1:**
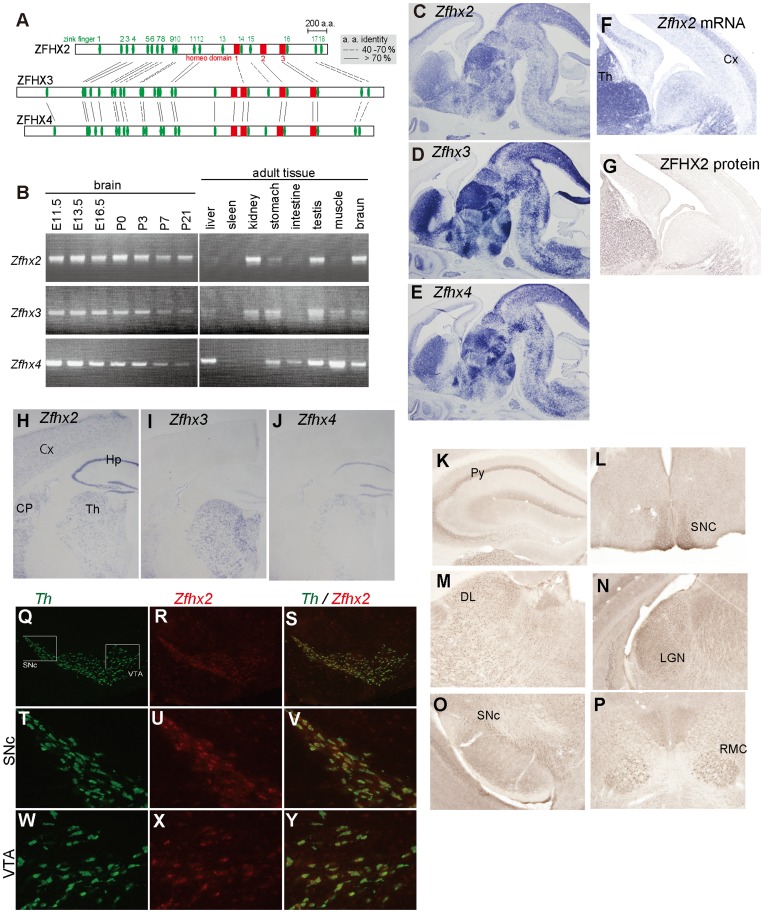
Structure and expression of mouse *Zfhx2*. (A) Structure of ZFHX2 and related proteins. ZFHX2 is a protein of 2562 amino acids containing 18 zinc fingers (green ovals) and three homeodomains (red squares). (B) *Zfhx2*, *Zfhx3*, and *Zfhx4* mRNA detected by semi-quantitative RT-PCR in various RNA sources. Note that cDNAs were amplified for 30 cycles for brains of different developmental stages, whereas for 35 cycles for various adult tissues. (C–E) Expression of *Zfhx2* (C), *Zfhx3* (D), and *Zfhx4* (E) mRNA in the parasagittal sections of an E15.5 mouse brain. These three genes were expressed in substantially similar patterns with the highest expression level of *Zfhx3*. (F, G) Expression of *Zfhx2* mRNA (F) and the ZFHX2 protein (G) was compared on adjacent coronal sections of an E15.5 brain. mRNA expressed in the thalamic region (Th) was translated, whereas mRNA expressed in the cerebral cortex (Cx) was not translated: this situation made the expression patterns of ZFHX2 and ZFHX3 more alike in the protein level than in the mRNA level. (H–J) Expression of *Zfhx2* (H), *Zfhx3* (I), and *Zfhx4* (J) mRNA in the coronal sections of an adult brain. Cerebral cortex (Cx), hippocampus (Hp), thalamus (Th), caudate putamen (CP). Expression levels of all three genes were decreased compared with those in the embryonic brain, but *Zfhx2* maintained higher level of expression than the others. (K–P) Expression of ZFHX2 protein in the adult brain. The pyramidal layer of hippocampus (K, Py), the suprachiasmatic nucleus (L, SCN), laterodorsal thalamic nucleus (M, LD), lateral geniculate nucleus (N, LGN), substantia nigra pars compacta (O, SNc), and magnocellular part of the red nucleus suprachiasmic (P, RMC). (Q–Y) Double-color in situ hybridization with *Zfhx2* and tyrosine hydroxylase (*Th*) probes. The *Zfhx2* mRNA (red) was highly expressed in the *Th* mRNA (green)-positive cells in the substantia nigra pars compacta (SNc). *Zfhx2* mRNA was coexpressed with *Th* mRNA also in the ventral tegmental area (VTA) at a slightly lower level.

Mammalian *Zfhx2* (also known as *zfh-5*) encodes a transcription factor of unknown function containing three homeobox domains and 18 Zn-finger motifs. *Zfhx2* is highly expressed in the developing mouse brain particularly in differentiating neurons and continues to be expressed throughout adulthood at a low level [1]. Two other phylogenically related genes, *Zfhx3* (*atbf1*) and *Zfhx4* (*zfh-4*), have been identified in the *Zfhx* family [2], the members of which contain three to four homeobox domains and 18 to 23 Zn-finger motifs (Fig. 1A). *Zfhx3* is expressed in the developing brain in a manner dependent on neuronal differentiation [3] and was also reported to be relevant in the malignancy of some classes of cancer [4], [5]. *Zfhx4* is a candidate gene causing congenital bilateral isolated ptosis [6]. Although these three genes are expressed in substantially similar patterns in the developing brain (Figs. 1C–E), common functional features have not been clarified.

Previously, we found that the antisense strand of *Zfhx2* is also expressed in the mouse brain in a manner complementary to the expression of *Zfhx2* mRNA. Although most neurons express *Zfhx2* mRNA immediately after their final mitosis, several types of neuron (e.g., granule cells in the olfactory bulb and pyramidal and granule cells in the hippocampus) express antisense RNA prior to *Zfhx2* mRNA during the early phase of their differentiation. By generating a gene-targeting mouse line in which *Zfhx2* sense RNA is expressed but not antisense RNA, we showed that this antisense RNA has a negative regulatory role in the expression of *Zfhx2* mRNA. These observations suggest that the ZFHX2 protein might have a role in a particular step of neuronal differentiation, and in some types of neuron, this step might be delayed by the expression of antisense RNA [1]. The specific function of ZFHX2, however, remains to be revealed.

To elucidate the function of ZFHX2, we have generated a *Zfhx2*-deficient mouse line. Although the production of the ZFHX2 protein is completely abolished in the homozygous mutant mice, the mice appear grossly normal and healthy. No anatomical abnormality has been observed in the mutant mouse brain so far examined. We hence subjected the *Zfhx2*-deficient mice to a comprehensive battery of behavioral tests [7], [8] to explore the physiological function of ZFHX2 in the nervous system. The homozygous *Zfhx2* deficient mice showed several behavioral abnormalities particularly in emotional aspects, such as a depression-like phenotype. In this paper, we report the behavioral phenotypes of the *Zfhx2*-deficient mice and discuss their possible relevance to human psychiatric disorders.

## Results

### Expression Profile of *Zfhx2*

The expression profile of *Zfhx2* mRNA and the ZFHX2 protein analyzed by reverse-transcriptase (RT)-PCR, in situ hybridization, and immunohistochemistry was summarized in Figure 1, together with those of the related genes, *Zfhx3* and *Zfhx4*. All three genes were expressed in various structures of the developing brain, and continued to be expressed throughout adulthood at lower expression levels. The expression profiles of these genes will be discussed again in the later section.

### General Characteristics of *Zfhx2*-deficient Mice

The *Zfhx2*-deficient mice appear grossly normal and healthy. No anatomical abnormality has been observed in the mutant mouse brain so far examined (data not shown). We subjected the *Zfhx2*-deficient mice and their wild-type littermates to a comprehensive battery of behavior tests to determine whether *Zfhx2* deficiency causes behavioral alternations (summarized in Table 1). The physical and neurological characteristics (i.e., body weight, body temperature, state of whiskers and fur, neurological reflexes, and muscle strength) were normal in the *Zfhx2*-deficient mice as compared with the control mice (Figs. S1A–D).

**Table 1:** List of behavioral tests.

Group	Test	Age *	Graphs	Descriptions **	Phenotypes of mutant mice suggested
1^st^	General health/neurological screen	11	Figs. S1A–D	Results-2	
	Light/dark transition	11	Figs. 3C–F	Results-3	hypoactivity in novel environments
	Open field	12	Figs. 2A–E	Results-3	hyperactivity, hypoactivity in novel environments, unusual anxiety
	Elevated plus maze	12	Figs. S2A–D	Results-6	
	Hot plate	12	Fig. S1F	Results-6	
	Social interaction (novel environment)	13	Figs. S3A–D	Results-6	
	Rotarod	13	Fig. S1E	Results-6	
	Social interaction (Crawly version)	14	Figs. S3E–H	Results-6	
	Prepulse inhibition/startle response	14	Figs. 5A, B	Results-6	
	Porsolt forced swim	15	Figs. 4A, B	Results-4	depression-like behavior
	Gait analysis	15	Figs. S4A, B	Results-6	
	Barnes maze	24	Figs. 5A–C	Results-5	
	Cued and contextual fear conditioning	27	Figs. S2E–G	Results-6	
	Tail suspension	29	Fig. 4C	Results-4	depression-like behavior
	24-h homecage monitoring	48	Figs. 3A, B	Results-3, 6	hyperactivity
2nd	General health/neurological screen	12			
	Light/dark transition	13	Fig. S6D	Results-3	hypoactivity in novel environments
	Open field	14	Figs. S6A, B	Results-3	hyperactivity, hypoactivity in novel environments, unusual anxiety
	24-h homecage monitoring, Circadian rhythm	24	Figs. S5A, B, S6C	Results-3, 6	hyperactivity

* ; Age (weeks old) of the youngest animals of the group at the start of the test. The oldest animals are 3 weeks (1st group) or 4 weeks (2nd group) older than the age indicated.

** ; Sections in the text where the results are described. Subsections of Results are; 2, General characteristics of *Zfhx2*-deficient mice; 3, Locomotor activity and anxiety-like behavior; 4, Depression-like behavior; 5, Sensorimotor gating; 6, Spatial reference memory; 7, Other behavioral tests.

### Locomotor Activity and Anxiety-like Behavior

**Figure 2:**
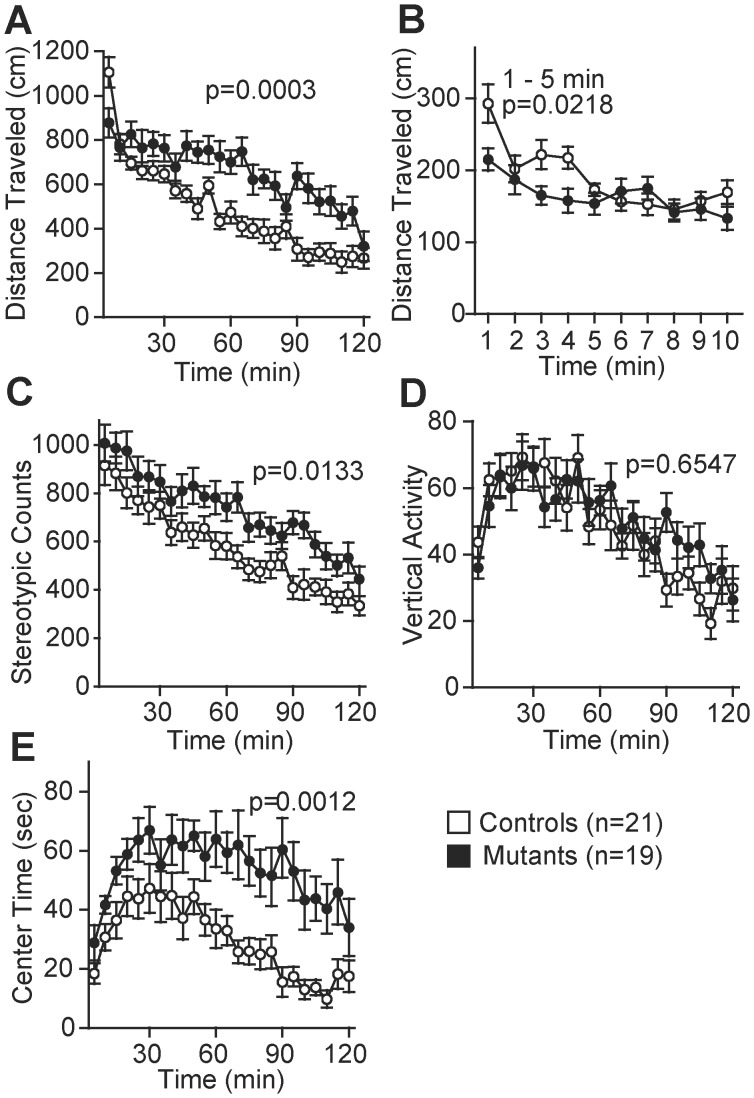
Locomotor activity measured in open field test. Distance traveled (A, B), stereotypic behavior (C), vertical activity (D), and time spent in the center area (E). Measurements are blocked in 5 min (A, C, D, and E) or in 1 min (B, shown only for the first 10 min). The *Zfhx2*-deficient mice were generally hyperactive but were hypoactive in the initial period in the open field.

**Figure 3:**
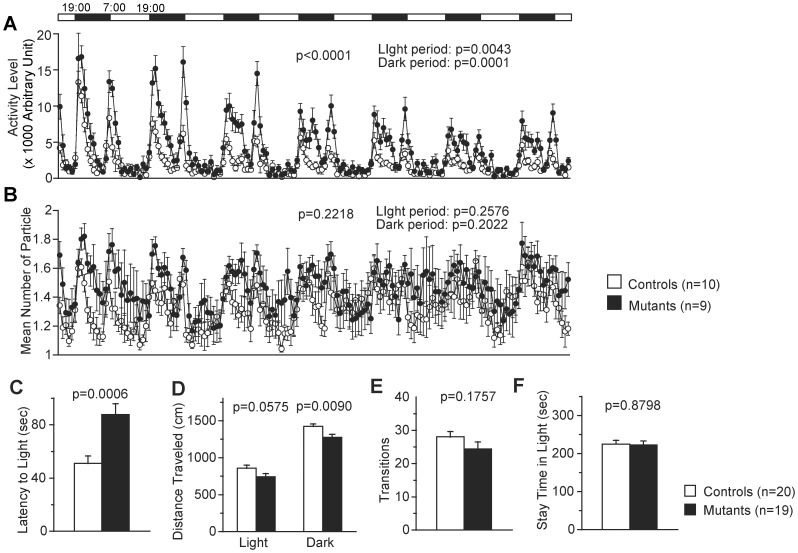
24-h homecage monitoring test and light/dark transition test. (A, B) 24-h homecage monitoring test: two mice of the same genotype were housed in a single cage and continuously monitored by a video camera. The activity level of the mutant mice was significantly higher (A), but the mean number of particles, which represented the social interaction level between cagemates, was not significantly different (B). Top bar indicates light/dark cycle. (C–F) Light/dark transition test: latency to enter the light chamber for the first time was significantly longer in the mutant mice (C), the mutant mice traveled shorter distances particularly in the dark chamber (D), no significant difference was observed in the number of transitions between the light and dark chambers (E) or in the time spent in the light chamber (F).

In the open field test, the distance traveled (*F*_1,38_ = 15.999, *p* = 0.0003, Fig. 2A) and stereotypic counts (*F*_1,38_ = 6.737, *p* = 0.0133, Fig. 2C) of the mutant mice during the entire 120-min period were significantly higher than those of the control mice suggesting a hyperactive phenotype of the *Zfhx2*-deficient mice. This phenotype was confirmed by the activity level measured in the 24-h homecage monitoring test (light period, *F*_1,17_ = 10.828, *p* = 0.0043; dark period, *F*_1,17_ = 25.235, *p* = 0.0001; total, *F*_1,17_ = 25.645, *p*<0.0001, Fig. 3A). In the open field, however, the pattern of temporal change in activity of the mutant mice was different from that of the control mice (genotype × time interaction, *F*_23,874_ = 3.877, *p*<0.0001). In the first 5 min, the mutant mice were less active than the control mice (*F*_1,38_ = 5.724, *p* = 0.0218, Fig. 2B). This observation was consistent with the following two results of the light/dark transition test: the mutant mice demonstrated a longer latency to enter into the light chamber for the first time (*F*_1,37_ = 14.015, *p* = 0.0006, Fig. 3C) and traveled a shorter distance in 10 min than the control mice, although the difference in the distance traveled in the light chamber did not reach a statistically significant level (light, *F*_1,37_ = 3.842, *p* = 0.0575; dark, *F*_1,37_ = 7.599, *p* = 0.0090, Fig. 3D) nor was observed a significant difference in the number of transitions between the light and dark chambers (Fig. 3E). Taken together, the *Zfhx2*-deficient mice were generally hyperactive, although they were specifically less active immediately after being transferred to novel environments.

In the open field test, the time spent in the center area by the mutant mice was significantly longer than that by the control mice (*F*_1,38_ = 12.275, *p* = 0.0012, Fig. 2E), which may be related to an abnormal anxiety-like behavior. On the other hand, no significant difference was observed in the time spent in the light compartment in the light/dark transition test, which is another anxiety-related measurement, between the mutant and control mice (Fig. 3F) (see Discussion).

Finally, using another group of animals, we repeated the light/dark transition test, open field test, and 24-h homecage monitoring test (Fig. S6) and confirmed these phenotypes of the *Zfhx2*-deficient mice, namely, the hypoactivity during the initial period after being transferred into novel environments and the hyperactivity in familiar environments, together with the phenotype of spending longer time in the center area of the open field.

### Depression-like Behavior

**Figure 4:**
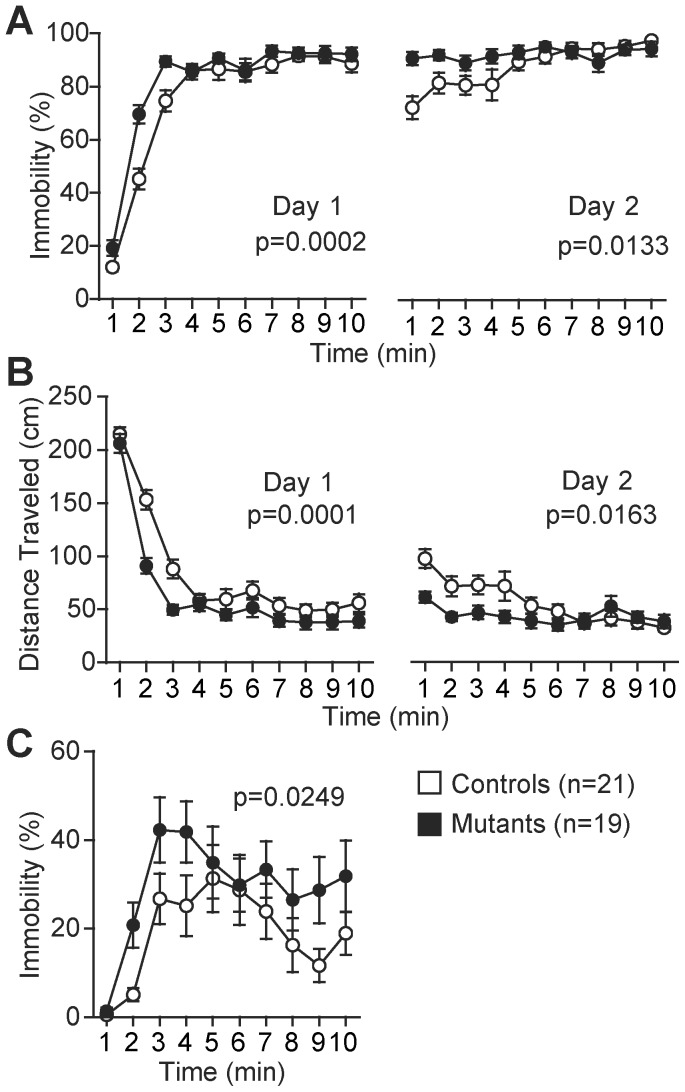
Enhanced depression-like behavior of *Zfhx2*-deficient mice. (A, B) Porsalt forced swim test: the percentage of time in immobile posture of the mutant mice was significantly higher (A) and the distance traveled by the mutant mice was significantly shorter (B) than those of the controls. (C) Tail suspension test: the percentage of time in immobile posture of the mutant mice was significantly higher than that of the control mice.

In the Porsolt forced swim test, the *Zfhx2*-deficient mice showed an immobile posture, which is considered as a reflection of a state of behavioral despair and commonly used as an index for evaluating depression-like behavior, for a significantly longer time than the control mice did (day 1, *F*_1,38_ = 17.343, *p* = 0.0002; day 2, *F*_1,38_ = 6.739, *p* = 0.0133, Fig. 4A). The distance traveled by the mutant mice was also significantly shorter than that by the control mice (day 1, *F*_1,38_ = 18.797, *p* = 0.0001; day 2, *F*_1,38_ = 6.322, *p* = 0.0163, Fig. 4B). In addition, the mutant mice showed a significantly increased immobility in the tail suspension test (*F*_1,38_ = 5.453, *P* = 0.0249, Fig. 4C). These results suggest that the *Zfhx2*-deficient mice showed an enhanced depression-like phenotype.

### Spatial Reference Memory

**Figure 5:**
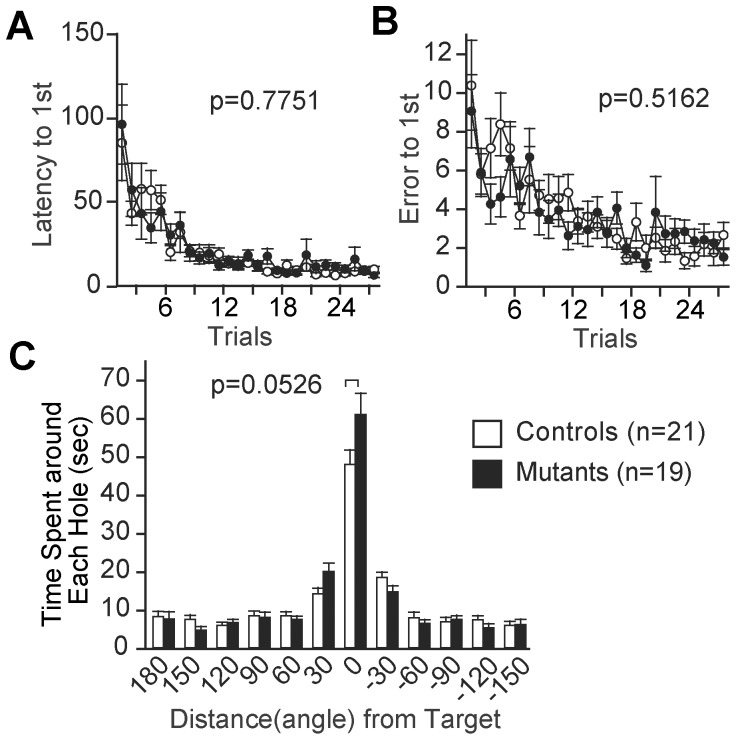
Spatial reference memory. Barnes maze test was performed to assess spatial reference memory. There was no significant difference in latency (A) and number of errors (B) to reach the target hole during the training period. (C) Probe trial conducted 1 day after the last training session. The results were analyzed using two-way ANOVA followed by Fisher’s PLSD post hoc test. A significant difference was found in the effect of (genotype) × (hole position ( = distance from target)) interaction (*F*_11,418_ = 2.989, *p* = 0.0008), although the difference of the time spent around the target did not reach a statistically significant level (genotype effect, *F*_1,38_ = 4.003, *p* = 0.0526).

To assess spatial reference memory, we performed the Barnes circular maze test, which resembles the Morris water maze test, using a white circular board instead of a milk pool. The *Zfhx2*-deficient mice had no defect in the memory acquisition process, judging from the latency and number of errors before the first time they reached to the target where an escape box was attached (Figs. 5A and B). In the probe test (exploration on the same circular board without an escape box attached for 3 min) conducted on the next day of the last training session (Fig. 5C), both mutant mice and control mice spent significantly longer time around the target hole than around the others (the effect of hole position ( = distance from the target in Fig. 5C), *F*_11,240_ = 56.229, *p*<0.0001 for controls, *F*_11,216_ = 59.340, *p*<0.0001 for mutants), suggesting that mice of both genotypes remembered the position of the target. However, there was a significant difference in the effect of (genotype) × (hole position) interaction (*F*_11,418_ = 2.989, *p* = 0.0008), i.e., the mutant mice and control mice preferred the target hole at different degrees. This result may suggest an increased perseveration tendency of the *Zfhx2*-deficient mice, although the difference of the time spent around the target did not reach a statistically significant level (genotype effect, *F*_1,38_ = 4.003, *p* = 0.0526).

### Other Behavioral Tests

Besides the tests described above, we also conducted the following behavioral tests: the elevated plus maze test, social interaction test in a novel environment, Crawley version social interaction test, rotarod test, hot plate test, startle response/prepulse inhibition test, cued and contextual fear conditioning test, gait analysis, and circadian rhythm analysis. Results of these behavioral tests are presented as supporting information (Figs. S1, S2, S3, S4, and S5).

In the startle response/prepulse inhibition test, the response of the mutant mice to sound stimuli was greater than that of the control mice (*F*_1,38_ = 6.095, *p* = 0.0182, Fig. S2H) and the percentage of inhibition of the response to a 120-dB stimulus by a low intensity ‘prepulse’ of the mutant mice was lower than that of the controls (*F*_1,38_ = 4.917, *p* = 0.0326, Fig. S2I). Although the differences are not significant enough considering multiplicity of measurements done on the first group of mice, these results suggest that the sensorimotor gating might be impaired in the *Zfhx2*-deficient mice. In the gate analysis, the mutant mice showed slightly longer durations of the ‘swing’ phase, indicating possibility of subtle defects in the limb muscles and/or joints of the mutant mice (Fig. S4). In the other tests, significant differences in results were not observed between the *Zfhx2*-deficient mice and control mice. In addition, no significant difference was observed in the mean number of particles in the 24-h homecage monitoring test, which was a measurement of the social interaction behavior, between the mutant and control mice (Fig. 3B).

## Discussion

Mammalian ZFHX2 is a large transcription factor (2562 amino acids, 273 kD) expressed mainly in the brain. To elucidate its function, we conducted a comprehensive battery of behavioral tests on the *Zfhx2*-deficient mice. Here, we report that *Zfhx2* deficiency caused several behavioral abnormalities in mice, particularly in the emotional aspects.

The open field test (Fig. 2A) and 24-h monitoring homecage test (Fig. 3A) clearly showed that the *Zfhx2*-deficient mice were generally hyperactive. They were, however, hypoactive during the initial period in the open field (Fig. 2B). Similar tendency was also observed in other genetically modified mice, i.e., hyperactivity of the mutant mice was less prominent during the initial period in the open field than during the later period (*DAT* knockdown [9], *CN* knockout [10], *GSK3β* transgenic [11], *Disc1* dominant-negative [12], *Dtnbp1* deletion [13]). In some reports, these observations were explained as a habituation defect [9], [11], [13]. In the *Zfhx2*-deficient mice, however, we assume that the exploratory motivation of a novel environment might be reduced because they were less active than the control mice immediately after being transferred to a novel environment. A longer latency to enter the light compartment in the light/dark transition test (Fig. 3C) supported this idea although further analysis remains to be carried out.

The Porsolt forced swim test and tail suspension test are representative behavioral tests commonly used for assessing depression-like behavior in rodents [14], [15]. In both tests, the *Zfhx2*-deficient mice showed the immobilized posture, which is considered as a reflection of their state of behavioral despair or ‘helplessness’, for longer time than the control mice (Fig. 4). These observations indicated that *Zfhx2* deficiency caused the enhanced depression-like phenotype. The *Zfhx2*-deficient strain may serve as a novel model of depression, although several questions remain to be resolved including relationships among various phenotypes observed in the mutant mice.

The anxiety-related phenotype of the *Zfhx2* mutant mice was complicated. In general, the center area of the open field apparatus, the light chamber of the light/dark transition box, and the open arms of the elevated plus maze are more aversive areas for the mice than the peripheral area, the dark chamber, and the closed arms, respectively. Thus, the measurements of the time spent in these aversive areas are considered to reflect the anxiety level of the mice. The *Zfhx2* mutant mice, however, showed inconsistent results among these measurements. The time spent by the mutant mice in the center area of the open field was significantly longer than that spent by the control mice throughout the test period (Fig. 2E), which indicated a reduced anxiety-like phenotype of the *Zfhx2*-deficient mice. In contrast, the time spent in the light chamber of the light/dark transition box (Fig. 3F), the number of entries into and the time spent in the open arms of the elevated plus maze (Figs. S2B, D) were not significantly different between the mutant and control mice. As discussed previously, ‘anxiety’ is not a single phenomenon, rather, it has a multidimensional nature [16], [17]. We presume that each test that we conducted may have assessed separate aspects of the anxiety-related phenotype of the *Zfhx2* mutant mice.

In the wild-type mouse, *Zfhx2* was highly expressed in the developing brain and continued to be expressed throughout adulthood at a lower expression level (Figs. 1B, C, and H). It is not clear, however, whether abnormalities observed in the *Zfhx2*-deficient mice were caused by developmental defects or malfunction of the mature brain (or both). It is possible that *Zfhx3*, a gene phylogenically related to *Zfhx2*, compensated for the deficiency of *Zfhx2* in the developing mutant brain because *Zfhx3* was expressed in a similar pattern to and at a higher level than *Zfhx2* in the wild-type brain (Figs.1C–G). As the brain matures, however, the expression level of *Zfhx3* quickly decreased and finally became much lower than that of *Zfhx2* in the adult brain (Figs. 1B, H–J). Hence, the effect of *Zfhx2* deficiency may appear more clearly in the adult nervous system.

In the adult brain, the highest expression level of the ZFHX2 protein was observed in the pyramidal cell layer of the hippocampus, the suprachiasmatic nucleus, laterodorsal thalamic nucleus, lateral geniculate nucleus, substantia nigra pars compacta, and magnocellular part of the red nucleus (Figs. 1K–P). Dopaminergic neurons in the substantia nigra pars compacta, one of the major dopaminergic cell sources, expressed *Zfhx2* mRNA at a high level, and dopaminergic neurons in other regions such as the ventral tegmental area also expressed the *Zfhx2* mRNA at a slightly lower level (Figs. 1Q–Y). It is possible that the loss of ZFHX2 may cause some impairment in the dopaminergic neurons, which may result in disturbance of the dopaminergic inputs into various parts of the brain. Further investigation may clarify the relationship of *Zfhx2* with the dopaminergic system and also with other monoaminergic systems, which may underlie the behavioral phenotypes of the *Zfhx2*-deficient mice.

In this study, we found that *Zfhx2* deficiency caused multiple behavioral abnormalities in mice, namely, hyperactivity, enhanced depression-like behavior, and anxiety-related abnormalities. In addition to these major phenotypes, the *Zfhx2*-deficient mice may have a reduced motivation to explore a novel environment. It is noteworthy that the *Zfhx2*-deficient mice appeared quite normal with regard to general cognitive abilities, such as motor learning (rotarod test, Fig. S1E), reference memory (acquisition process of the Barnes maze test, Figs. 5A and B) and context and episodic-like memory (fear conditioning test, Figs. S2E–G), in contrast to the wide range of impairments observed in emotional aspects. Despite these behavioral phenotypes of the *Zfhx2*-deficient mice, functional relationships have not been known between *Zfhx2* and other genes related to neuropsychiatric disorders, such as serotonin-related genes, dopamine-related genes, *calcineurin* and *bdnf*. Thus, further analysis of the function of *Zfhx2* may provide a key to the clarification of novel aspects of the pathophysiology of these neuropsychiatric disorders.

## Materials and Methods

### Ethics Statement

All animal procedures were approved by the Institutional Animal Care and Use Committee of National Institutes of Natural Sciences (approval numbers: 10A173 and 11A102) or the Animal Care and Use Committee of Kyoto University Graduate School of Medicine (approval number: MedKyo09539) and performed in accordance with their guidelines.

### Animals

Generation of the *Zfhx2*-deficient mouse line was described by Komine et al. [1], where *Zfhx2* and the *Zfhx2*-deficient mouse line were referred to as *zfh-5* and the ORF replacement line, respectively. Heterozygous mutant mice, which had been backcrossed on a C57B6/J background for at least 12 generations, were intercrossed and the resulting homozygous and wild-type males were used for behavioral tests. Behavioral test battery was started after animals reached 11 weeks of age. Four mice were housed in one cage in a room with a 12-hr light/dark cycle with access to food and water ad libitum. Behavioral tests were performed between 9:00 and 18:00.

### RT-PCR

Reverse transcription (RT) was performed with 1 µg total RNA from mouse brains of various developmental stages and various adult tissues using SuperScript II (Invitrogen), and 1/100 of the resulting cDNA was amplified using ExTaq (Takara) for 30 cycles (E11.5 - P21 brains) or 35 cycles (adult tissues). Sequences of the primers used are available on request.

### In situ Hybridization

Embryonic brains fixed with 4% paraformamide or fresh adult brains were cryosectioned and in situ hybridization was performed as previously described [1], [18]. Briefly, after postfixation in 4% paraformaldehyde, acetylation and prehybridization, sections were hybridized with a digoxigenin (DIG)-labeled RNA probe. Following posthybridization washing, DIG-probes were detected using an alkaline-phosphatase (AP)-conjugated anti-DIG antibody and BCIP/NBT as an AP substrate.

For the double-color detection of *Zfhx2* and tyrosine hydroxylase (*Th*), a mixture of four fluorescein (FITC)-labeled *Zfhx2* antisense probes (in order to improve detection sensitivity) and a DIG-labeled *Th* probe was used. DIG-probes were detected using an AP-conjugated anti-DIG antibody and the HNPP Fluorescein Detection Set (Roche), and FITC-probes were detected using a horseradish-peroxidase (HRP)-conjugated anti-FITC antibody, amplified with the TSA-Plus DNP system (PerkinElmer), and finally visualized using an Alexa Fluor 488-conjugated anti-DNP antibody (Molecular Probes). Information about regions of the genes (*Zfhx2*, *Zfhx3*, *Zfhx4*, and *Th*) used for probes is available on request.

### Immunohistochemistry

A rabbit antibody raised against an N-terminal portion (amino acid 11-360) of ZFHX2 protein was used to examine localization of ZFHX2 protein. Staining was performed on paraformamide-fixed cryosections by a standard method using vecstain elite ABC kit (Vector laboratories) and DAB as a HRP substrate.

### Behavioral Tests

We performed a battery of behavioral tests consisting of the following experiments (listed in the order conducted): the general health and neurological screening test, light/dark transition test, open field test, elevated plus maze test, hot plate test, social interaction in novel environment test, rotarod test, startle response/prepulse inhibition test, Porsolt forced swim test, Crawley version social interaction test, gait analysis, Barnes maze test, cued and conditional fear conditioning test, tail suspension test and 24-h homecage monitoring test. Results of all tests of the battery are summarized in the supporting table (Table S1). Details of the experiments not mentioned below are described elsewhere [8], [19], [20]. Raw data from the behavioral tests are available in the mouse phenotype database (http://www.mouse-phenotype.org/).

### Open Field Test

Each mouse was placed in the corner of the open field apparatus (40×40×30 cm; Accuscan Instruments, Columbus, OH). The chamber of the test was illuminated at 100 lux. Distance traveled, vertical activity (rearing measured by counting the number of the times the high-position photobeam was interrupted), time spent in the center area (20×20 cm), and counts of stereotypical behaviors (counted when the same beam was repeatedly interrupted) were recorded. Data were collected for 120 min.

### Light/dark Transition Test

The apparatus consisted of a cage (21 cm×42 cm×25 cm) divided into two chambers of equal sizes by a partition with a door (O’HARA & Co., Tokyo, Japan). One chamber was brightly illuminated (390 lux), whereas the other chamber was dark (2 lux). Mice were placed into the dark chamber and allowed to move freely between the two chambers with the door open for 10 min. The latency to the first entry to the light chamber, distance traveled, total number of transitions between chambers, and time spent in each chamber were recorded and analyzed using Image LD software. Details of the experiment are described in Takao and Miyakawa [21].

### Porsolt Forced Swim Test

The apparatus consisted of a Plexiglas cylinder (20 cm height×10 cm diameter) placed in the center of an opaque plastic chamber (30 cm×30 cm×30 cm). The cylinder was filled with water (23°C) up to a height of 7.5 cm. Mice were placed into the cylinder, and their behavior was recorded for 10 min and analyzed using Image PS software. Retention test trials were administered 24 hours after the first trials.

### Tail Suspension Test

Mice were suspended from the ceiling of an opaque plastic chamber (30 cm×30 cm×30 cm) by an adhesive tape placed <1 cm from the tip of the tail. Their behavior was recorded for 10 min, and image data were analyzed automatically using Image TS software.

### Barnes Circular Maze Test

The Barnes circular maze test was conducted on a white circular board, 100 cm in diameter, with 12 holes equally spaced around the perimeter (O’HARA & Co., Tokyo, Japan). The circular board was elevated 75 cm from the floor. A black Plexiglas escape box (17 cm×13 cm×7 cm), which had paper bedding on its bottom, was located under one of the holes. The hole above the escape box represented the target, analogous to the hidden platform in the Morris water maze task. The location of the target was consistent for a given mouse, but was randomized across mice. The maze was rotated daily, with the spatial location of the target unchanged with respect to the visual room cues, to prevent a bias based on olfactory or proximal cues within the maze. Mice were placed at the center of the circular board, and the latency, number of errors and distance traveled to reach the target hole were recorded. Three trials per day were conducted for nine days. One day after the last training, a probe trial, in which mice navigated on the same circular board without the escape box for 3 min, was conducted and the time spent around each hole was recorded.

### 24-hour Monitoring in Home Cage

A system that automatically analyzes the locomotor activity of mice in the home cage was used. The system contains a home cage (29 cm×18 cm×12 cm) and a filter cage top, separated by a 13-cm-high metal stand containing an infrared video camera, which is attached to the top of the stand. Two mice of the same genotype that had been housed separately were placed together in a home cage. Their locomotor activity and social behavior were monitored for 1 week. Outputs from the video cameras were fed into a computer and images from each cage were captured at a rate of one frame per second. Distance traveled was measured automatically using Image HA software. Social interaction was measured by counting the number of particles detected in each frame: two particles indicted that the mice were not in contact with each other, and one particle (i.e., the tracking software could not distinguish two separate bodies) indicated contact between the two mice.

Using the same system, circadian rhythms of locomotor activity were also analyzed. Each mouse was individually housed for 2 weeks under a 12-hour light-dark cycle condition (LD) and then for 2 weeks in constant darkness (DD). Distance traveled was automatically measured and analyzed.

### Data Analysis

Behavioral data were obtained automatically using applications based on the public domain NIH Image program and Image J program, with modifications for each test by the authors (available through O’HARA & Co., Tokyo, Japan). Statistical analysis was conducted using StatView (SAS Institute, Cary, NC). Data were analyzed by one-way or two-way ANOVA, or two-way repeated measures ANOVA. *F* and *p* values indicated in the text and graphs represent the effects of genotype unless otherwise noted. Values in graphs are expressed as mean ± SEM.

## Supporting Information

Figure S1 **No abnormality was observed in the physical characteristics of the** ***Zfhx2*** **-deficient mice.** Body weight (A), body temperature (B), grip strength (C), wire hang test (D), latency to fall in the rotarod test (E), and latency to the first response in the hot plate test (F).
(TIF)

Figure S2 **Elevated plus maze test, cued and context fear conditioning test, and startle response/prepulse inhibition test.** (A–D) Elevated plus maze test: no significant difference was observed in the number of entries (A), percent entries into open arms (B), distance traveled (C), and time spent on open arms (D). (E–G) Fear conditioning test: percent freezing in conditioning (E), context testing (F), and cued testing (G). Horizontal bars and arrows indicate tone and foot shock presentation, respectively. (H, I) Startle response/prepulse inhibition test: the amplitude of the startle response to the acoustic stimuli (H), and the percentage of prepulse inhibition of the startle response (I). p-values represent the effects of genotype using the sound level (H) or prepulse level (I) as repeated measures.
(TIF)

Figure S3 ***Zfhx2*** **-deficient mice showed normal performances in social interaction tests.** (A–D) Social interaction test in novel environment: total duration of contact (A), number of contacts (B), mean duration of contacts (C), and distance traveled (D). (E–H) Crawley version social interaction test: (E, F) Number of entries (G) and time spent (H) around a cage with a stranger mouse and an empty cage: no significant effect of (genotype) × (stranger/empty) interaction was observed. (I, J) Number of entries (I) and time spent (J) around a cage with a stranger mouse and a cage with a familiar mouse: no significant effect of (genotype) × (stranger/familiar) interaction was observed.
(TIF)

Figure S4 **Results of gait analysis.** The *Zfhx2*-deficient mice showed slightly longer durations of the ‘swing’ phase, indicating possibility of subtle defects in the limb muscles and/or joints.
(TIF)

Figure S5 **Circadian rhythm of locomotor activity.** (A) Representative double-plotted activity records from two control mice and two mutant mice. Data from the last 7 days under 12-h light-dark cycle condition (LD) and 16 days under constant dark condition (DD) are shown. (B) Circadian period length estimated from activity records under DD condition. The *Zfhx2*-deficient mice showed a normal period length.
(TIF)

Figure S6 **Additional behavioral tests.** The second group of animals (homozygous mutant males and their wild-type littermates) was subjected to the light/dark transition test, open field test, and 24-h homecage monitoring test to confirm the phenotypes observed in the first group of animals. (A, B) Open field test. Total distance traveled (A) and time spent in the center area (B) blocked in 5 minutes. In (A), genotype effect for the entire period, *F*_1,38_ = 0.751, *p* = 0.3916; for initial 30 min, *F*_1,38_ = 4.342, *p* = 0.044; for the last 30 min, *F*_1,38_ = 6.670, *p* = 0.0138; the effect of (genotype) × (time) interaction for the entire period, *F*_23,874_ = 6.388, *p*<0.0001. (C) Total distance traveled in the 24-h monitoring cage. Note that each mouse was separately housed in this experiment, whereas two mice were housed together in the experiment of Fig. 2. (D) Latency to enter the light chamber for the first time.
(TIF)

Table S1 Results of comprehensive battery of behavioral tests. Values are shown as means ± SEM. *F* and *p* values are effects of genotype, except *. *; effects of stranger/empty or stranger/familiar.
(XLS)
