# Supplementary figures and images for: Behavioral Abnormalities Observed in Zfhx2-Deficient Mice
Source: PLoS One. 2012 Dec 31;7(12):e53114. doi: 10.1371/journal.pone.0053114 (PMC3534046; doi:10.1371/journal.pone.0053114)

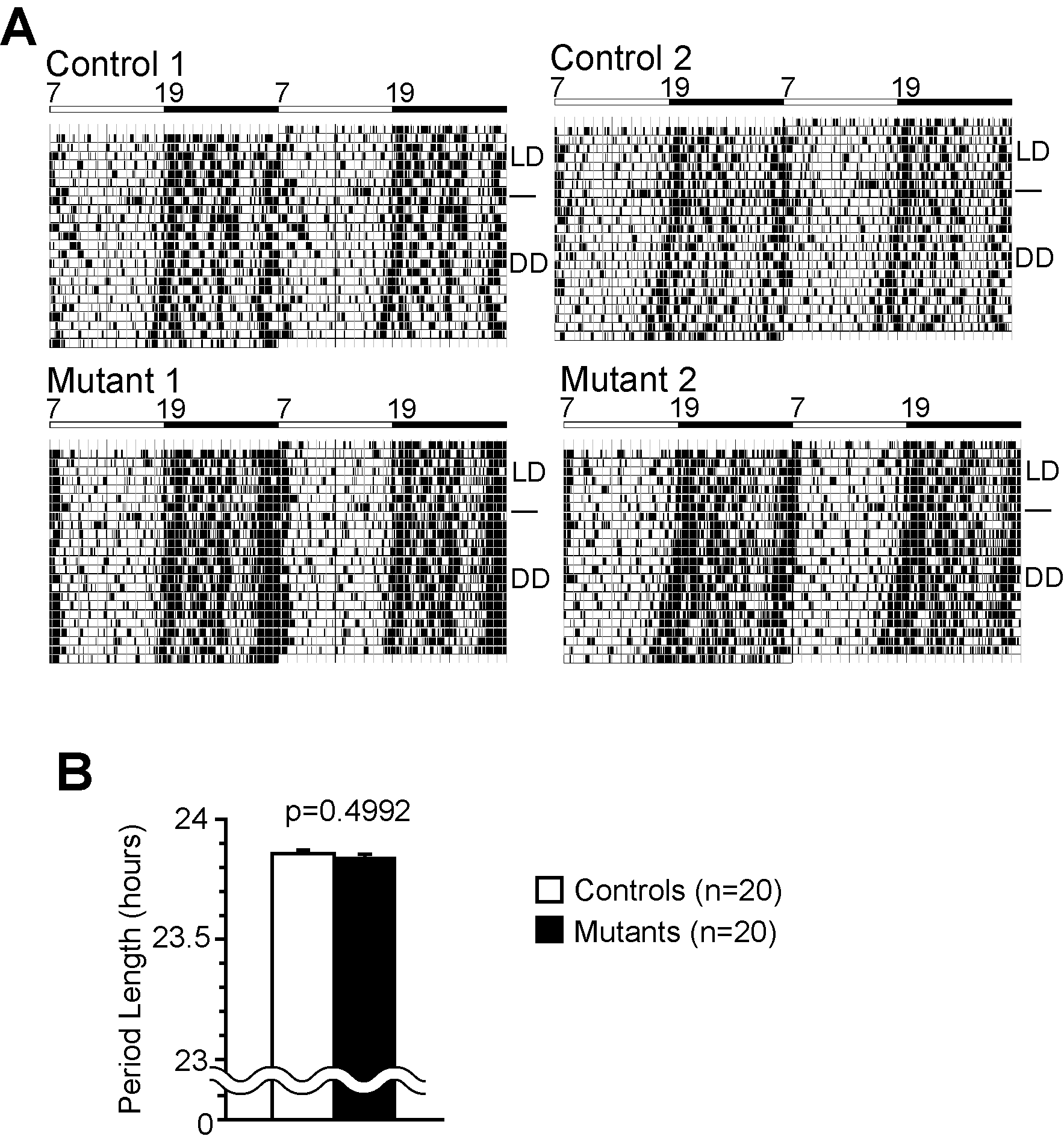

Supplement: Figure S5 — Circadian rhythm of locomotor activity. (A) Representative double-plotted activity records from two control mice and two mutant mice. Data from the last 7 days under 12-h light-dark cycle condition (LD) and 16 days under constant dark condition (DD) are shown. (B) Circadian period length estimated from activity records under DD condition. The Zfhx2-deficient mice showed a normal period length. (TIF) [file pone.0053114.s005.tif]
